# Accuracy Evaluation of Carotid-Femoral Pulse Wave Velocity Estimated by Smart Terminal Watch

**DOI:** 10.3389/fcvm.2022.893557

**Published:** 2022-07-22

**Authors:** Ningling Sun, Luyan Wang, Yang Xi, Hongyi Wang, Fan Yang, Yuanyuan Chen, Jing Liu, Yuxian Cui, Zhechun Zeng

**Affiliations:** ^1^Department of Hypertension, Peking University People’s Hospital, Beijing, China; ^2^Beijing Institute of Heart Lung and Blood Vessel Disease, Beijing Anzhen Hospital, Capital Medical University, Beijing, China

**Keywords:** cervical-femoral pulse wave velocity, arterial stiffness, smartwatch, accuracy, Complior arteriosclerosis analyzer

## Abstract

To evaluate the accuracy of the smartwatch in estimating carotid-femoral pulse wave velocity (cfPWV). A cohort of gender-matched volunteers aged 18–80 years were recruited. At the sitting and supine positions, cfPWV was measured alternately by smartwatch and CompliorAnalyse, for each participant, and nine sets of data were collected from each participant with a 60 s interval between measurements. The accuracy of cfPWV measurement for smartwatches was assessed using mean error (ME) and mean absolute error (MAE), while the consistency of the two methods was assessed using the Bland-Altman analysis and concordance class correlation. A total of 347 participants were enrolled. The mean cfPWV was 9.01 ± 2.29 m/s measured by CompliorAnalyse and 9.06 ± 1.94 m/s by smartwatch. The consistency correlation coefficient (CCC) was 0.9045 (95% CI 0.8853–0.9206), the ME was 0.046 ± 0.92, and the MAE was 0.66 (95% CI 0.59–0.73). Bland-Altman analysis showed that the error of 95% samples was in the range between −1.77 m/s and 1.86 m/s. The Kappa value of cfPWV greater than 10 m/s was 0.79, the area under the ROC curve was 0.97 (*P* < 0.001), sensitivity was 0.90, specificity was 0.93, positive predictive value was 0.83 and negative predictive value was 0.96. Smartwatch can accurately estimate cfPWV to evaluate arterial stiffness. This method is simple and feasible and is suitable for people to actively and early monitor vascular elasticity.

## Introduction

Arteriosclerotic disease is a sneaky, progressive condition. Patients will experience vascular functional disease first, followed by structural vascular disease. Clinically, patients may be asymptomatic and have increased arterial stiffness. Arteriosclerosis and atherosclerosis will worsen over time if the patient does not pay attention, leading to cardiovascular and cerebrovascular diseases (1–4). At present, detecting arterial stiffness requires specialized equipment in a hospital, which comes with many drawbacks, including high costs and lengthy wait times (going to the hospital for examination, appointment, registration, etc.). As a result, developing simple tools for detecting arteriosclerosis is critical. Arteriosclerosis can be prevented provided arterial stiffness is detected early enough (5). This study will start with active disease prevention, early detection, and early intervention in this research. As a result, some accurate and straightforward wearable devices that will assist in achieving this goal are required.

Huawei Smart Terminal has developed a mobile wearable smartwatch that can assess health status and has successfully launched it on the market. Multi-function sensors on the smartwatch collect the wearer’s ECG, pulse, and other data (6). The pulse wave velocity (PWV) of the artery is estimated using its built-in machine learning algorithm and linked with a smartphone APP to assess the stiffness of the artery. The smartwatch is currently the only portable and wearable smart mobile terminal watch in China to evaluate carotid-femoral pulse wave velocity (cfPWV). Although there have been watches or bracelets that can estimate arterial elasticity in the past (7), no watch can complete the detection of cfPWV as recommended by the European guidelines of hypertension (8) and the Chinese Guidelines for Prevention and Treatment of Hypertension (9). The cfPWV is one of the accepted indicators for evaluating vascular and target organ damage (8, 9). Although this smartwatch is already on the market, it remains unclear whether it can be accurately applied to clinical or real-world scenarios to provide personalized arterial elasticity health guidance. We initiated an evaluation of the accuracy of this smartwatch based on clinical needs to investigate whether this smart terminal watch can accurately complete the estimation of cfPWV. As a result, we established a project at Peking University People’s Hospital entitled “Research on Arteriosclerosis Detection and Mobile Terminal Verification,” with the project approval number 2019-Z-28. The topic of the project was the use of the Huawei smart terminal watch to estimate cfPWV and the measure of cfPWV by the French Complior arteriosclerosis analyzer as a reference device (gold standard) to assess the wearable mobile smartwatch’s accuracy in testing arterial stiffness.

## Materials and Methods

According to statistical requirements, at least 320 participants were recruited from the social population, with at least 100 participants from each of the three age groups, namely < 30 years old, 30–60 years old, and > 60 years old. This study was approved by the Ethics Committee of Peking University People’s Hospital, with the ethics approval number: 2019 PHB-196-01, and all participants filled in the informed consent form before participating in the study.

### Research Criteria

#### Inclusion Criteria

a.>18 years old, and gender is not limited;

b.Healthy participants with hypertension, diabetes, or dyslipidemia considered;

c.Participants with normal heart rhythm (no arrhythmia/pulse disturbance during measurement);

d.Participants who can cooperate with medical staff to conduct arteriosclerosis detection;

e.Participants who can voluntarily join the group and sign the informed consent.

#### Exclusion Criteria

a.Patients with a definite diagnosis of cardiovascular and cerebrovascular diseases;

b.People with mood disorders (anxiety, nervousness) and conditions that are not suitable for continuing arterial elasticity measurement;

c.People with peripheral arterial occlusive lesions of the left and right carotid arteries, upper extremity arteries, iliac arteries, and femoral arteries are unable to perform the PWV test;

d.Participants may be rejected for any reason;

e.The researcher believes that the candidate is ineligible for consideration.

### Research Methods

#### Basic Information Collection

Gender, age, height, weight, and medical history were all recorded as part of the patient’s general clinical information (disease history, treatment history). Each participant sat in the clinic for 15 min and had their heart auscultated for 2 min, according to the 2018 Chinese Guidelines for the Prevention and Treatment of Hypertension (9), to exclude arrhythmia. Choose an appropriate cuff based on the patient’s arm circumference and measure blood pressure three times using an electronic sphygmomanometer (OMRON HBP1300). If the difference between the blood pressure values measured each time is within 5 mmHg if it exceeds 5 mmHg, the number of measurements is increased, and the average value is calculated.

#### cfPWV Data Collection and Process

Following rigorous training, cardiovascular specialists and technicians at Peking University People’s Hospital measured nine sets of data for each subject. The specific operations are as follows: The researchers turned on the terminal smartwatch (model: HUAWEI WATCH GT 2 Pro ECG) from 8:00 to 10:00 in the morning to match the mobile operating system (Android or Hongmeng) *via* the Bluetooth protocol. Next, they installed and opened the matching APP on the phone.

After 10 min of rest, the participant’s left hand wears a smart terminal watch, and the right hand gently touches the collector on the watch. The participant’s electrocardiogram and photoplethysmography (ECG + PPG) data are collected to calculate the cfPWV (Huawei- Estimated-Carotid Femoral Artery-PWV). Each measurement time is 30 s, with each interval of 60 s. The data can be viewed and recorded using the corresponding mobile phone APP. The data is presented in HW-cfPWV units. If the difference between the three measurements is greater than 0.5 m/s, a measurement is added, and an average is calculated.

The participant takes a supine position, and the professional tester measures the distance between the left carotid artery and the femoral artery using the French Comparality Automatic pulse wave velocity measurement system, model number: CompliorAnalyse (also known as the Complior arteriosclerosis analyzer). Set the pressure receiver to two arteries and fine-tune it until the screen displays the correct pulse waveform. When measuring the participant’s 10 heart cycles, it is considered effective if the error range fluctuates within 5%. The measurement was carried out three times. Complior-cfPWV is used to represent the obtained data. If the difference between the three measurements is greater than 0.5 m/s, add one measurement and calculate the average value to complete the measurement.

In the lying position, the participant collects ECG + PPG information (measurement for 30 s) by wearing (left hand) the Huawei smart watch (measurement for 30 s). Simultaneously, the cfPWV is measured, and two measurements are alternately performed (three times alternately), each time the interval is 60 s.

In this study, we used the measurement results of Complioranalyse as a gold standard for cfPWV and compared the same research object using a cfPWV result measured by an intelligent terminal watch algorithm shown in Supplementary Figure 1.

### Statistical Analysis and Assessment Metrics

Statistical analysis were conducted in RStudio (version 2021.09.1 build 372, R version 4.12) and NCSS (version 15, NCSS, LLC). Continuous variables are described by mean ± standard deviation, and categorical variables are expressed as numbers (percentage).

**Concordance Class Correlation (CCC)** is used to evaluate the overall agreement between the testing device and the gold standard device.

**ME (Mean Error), MAE (Mean Absolute Error), MAPE (Mean Absolute Percentage Error)** are used to measure the specific error between the testing device and the gold standard device.

**Bland-Altman analysis**: Bland-Altman plot was used to visualize the characteristics of relative difference (testing device minus gold standard device). 95% LoA (limits of agreement) was used to determine the acceptable error range.

**Generalized linear models** are used to analyze the factors influencing the measurement error.

**Kappa, ROC curve analysis** are used to analyze the agreement and detection capability of the testing device in determining atherosclerosis (cfPWV > 10 m/s) with the gold standard device.

## Results

### The Basic Information of the Participant Included in This Study

A total of 347 participants (175 males, 172 females), including 243 healthy people (BMI < 24 kg/m^2^, no hypertension, diabetes, hyperlipidemia, etc.). Further, 104 patients suffering from hypertension, diabetes, and blood lipids were included. The average age of the participant was 51.88 ± 18.50 years old, with an average systolic pressure of 118.52 ± 15.46 mmHg, with an average diastolic pressure of 74.52 ± 9.15 mmHg, as shown in Table 1.

**TABLE 1:** Baseline characteristics of study participants (*N* = 347).

Item	Mean ± SD
Age (year)	51.88 ± 18.50
Sex (male) [n (%)]	175 (50.42)
Combined risk factors [n (%)]	104 (29.97)
Medications [n (%)]	100 (28.82)
BMI (kg/m2)	24.37 ± 3.54
SBP (mmHg)	118.52 ± 15.46
DBP (mmHg)	74.52 ± 9.15
Heart rate (bpm)	68.12 ± 10.49
cfPWV (m/s)*	9.01 ± 2.29

*cfPWV measured with Complior arteriosclerosis Analyzer Combined risk factors: hypertension, hyperlipids, hyperglycemia; medications: drugs treating for combined risk factors (e.g., hypoglycemia agents for patients with diabetes and so on).

### The Consistency Analysis of cfPWV Data Measured by Intelligent Terminal Watches Using Complior Analyze

#### The cfPWV (HW-cfPWV) Data Estimated by Huawei Watch Was Compared With the Distribution of the Complior-cfPWV Measurement Data

**FIGURE 1:**
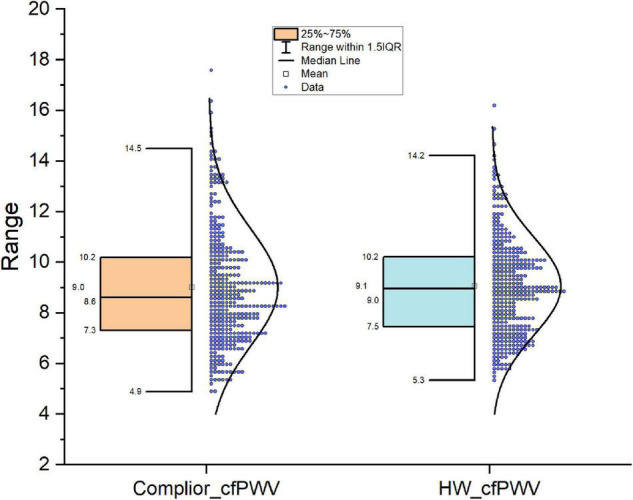
Comparison of distribution shape of the two cfPWV results measured with smart watch and Complier arteriosclerosis analyzer.

The mean of Complior-cfPWV was 9.01 ± 2.29 m/s, and the median was 8.61 m/s. The average value of the smartwatch HW-cfPWV was 9.06 ± 1.94 m/s, and the median was 8.90 m/s. The morphology of the two groups of data distributions, centralized trend indicators (mean, median), discrete trend indicators (standard deviation, quadrotes, extreme values), and other indicators are similar shown in Figure 1.

#### Consistency Analysis of HW-cfPWV and Compiler-cfPWV Results

**FIGURE 2:**
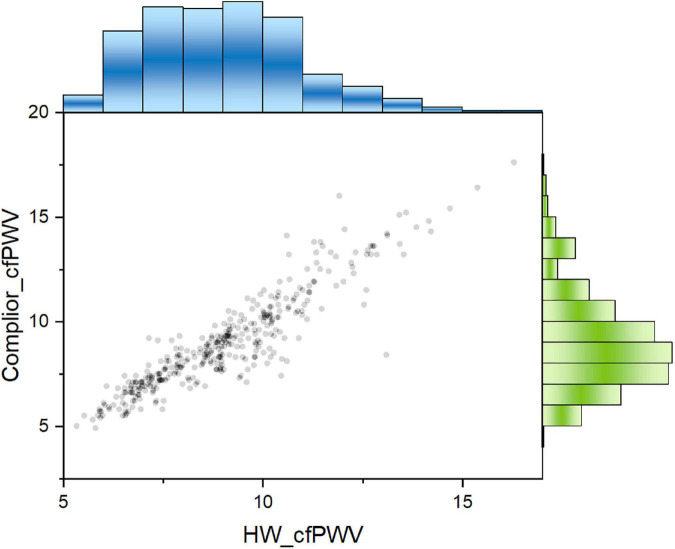
Two cfPWV values showed a significant positive correlation. Pearson correlation coefficient was 0.918. Blue columns: population distribution range of HW-cfPWV; Green columns: population distribution range of Complior-cfPWV.

The results showed that the Concordance Correlation Coefficient (CCC) is 0.9045, with a 95 percent confidence interval (CI) of 0.8853–0.9206 and a Pearson Correlation Coefficient (Pearson) of 0.9182, indicating that the two sets of data consistency are better (Figure 2).

#### The Correlation Between HW-cfPWV and Complior-cfPWV Was Then Layered

**FIGURE 3:**
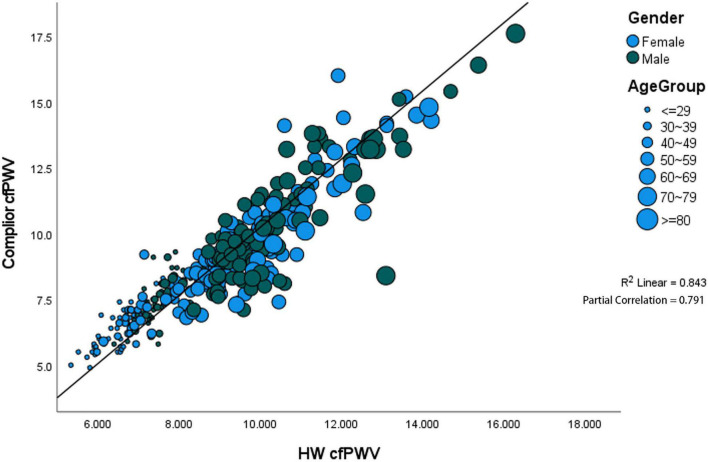
Significant positive correlation between gender and age. The partial correlation coefficient was 0.791.

The results show that the correlation coefficient of HW-cfPWV and Complior-cfPWV is high, with *P*-value < 0.0001 when age, gender, blood pressure, heart rate, hypertension, whether the participant takes medicine, and other layers are considered (Supplementary Table 1). After controlling for the aforementioned variables, the two data sets remain significantly correlated, with the partial correlation coefficient being 0.7911 (Figure 3).

#### Bland-Altman Analysis Between HW-cfPWV and Complior-cfPWV

**FIGURE 4:**
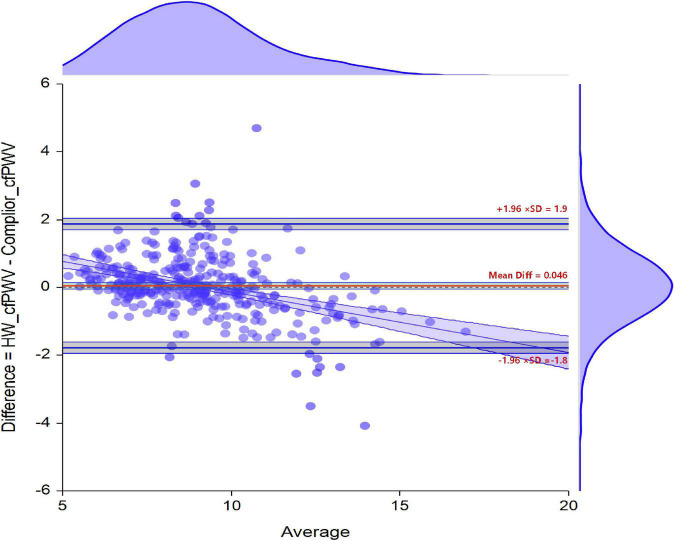
Bland-Altman graphical analysis of two types of cfPWV measurements.

The difference between the two sets of data was 0.046 (95% CI −0.0518 to 0.1440), and the B-A standard error (Me) was 0.046, the B-A standard deviation (STD) was 0.92, with the 95% sample measurement error distribution ranging from −1.77 m/s (95% CI −1.9395 to −1.6044) to 1.86 m/s (95% CI 1.6966 to 2.0316). The error is not a random distribution in the figure, and there is a linear relationship between the actual value and the error (Figure 4).

### Equivalent Analysis of HW-cfPWV and Complior-cfPWV

The deviation of the cfPWV level measured by both HW-cfPWV and Complior-cfPWV in 347 people was 0.046. The cfPWV measurements have passed the equivalent test of 1 m/s, regardless of whether the paired *t*-test or non-parametric method, Wilcoxon Signed-Rank Test was used, and the statistical power is 0.99. The results show that the cfPWV value calculated from the HW smartwatch is equivalent to the Complior-cfPWV equivalent (the mean error is within ± 1 m/s), as shown in Supplementary Tables 2, 3.

According to the International Artery Association Guide (10), the following criteria were considered for evaluating the accuracy of non-invasive pulse wave conduction speed measurement equipment (10): The mean error ≤ 0.5 m/s and the standard deviation ≤ 0.8 m/s is deemed to be EXCELLENT; mean error ≤ 1.0 m/s and standard deviation ≤ 1.5 m/s is considered to be ACCEPTABLE; mean error > 1.0 m/s or standard deviation > 1.5 m/s is deemed to be POOR. Comprehensive research results: the Me of B-A is 0.046; the STD of B-A is 0.92; the degree of detection of ± 1 m/s can determine the accuracy of the cfPWV obtained by the HW smartwatch algorithm to meet the ACCEPTABLE criteria.

### ROC Analysis of the Clinical Value of HW-cfPWV and Complior-cfPWV to Identify Arteriosclerosis

**FIGURE 5:**
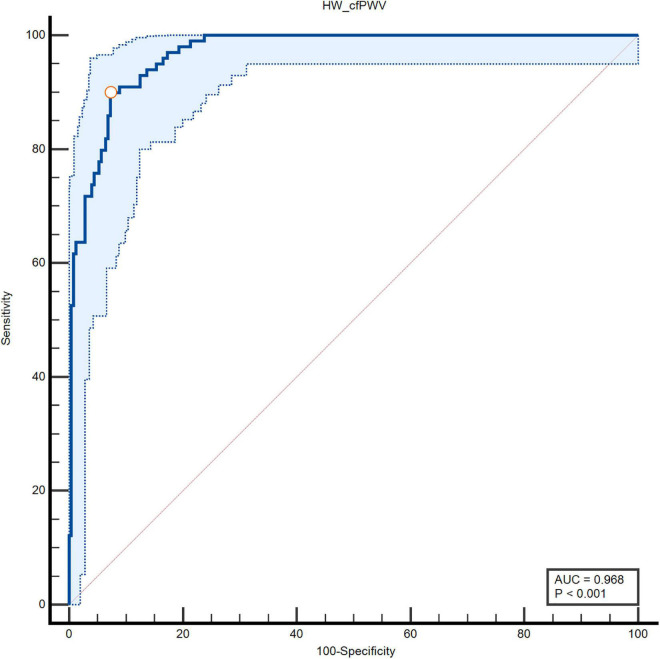
Specificity and sensitivity of area under the ROC curve of two types of cfPWV measurements.

The ROC curve of HW-cfPWV and Complior-cfPWV is 0.968, *P* < 0.001, the sensitivity is 0.899, the specificity is 0.927, indicating that the smartwatch measurement has a good ability to identify arteriosclerosis (cfPWV > 10 m/s), as shown in Figure 5 and Table 2.

**TABLE 2:** The diagnostic ability of smartwatch algorithm to judge arterial stiffness (cfPWV > 10 m/s).

	Value	95% CI Lower	95% CI Upper
Cut-off Point	> 9.94	>9.83	> 10.02
Sensitivity	0.8990	0.82	0.95
Specificity	0.9274	0.89	0.96
Positive predictive value	0.83	0.75	0.90
Negative predictive value	0.96	0.92	0.98
Macro F1 value	0.86	0.78	0.92

*CI, confidence interval.*

### Consistency Analysis of HW-cfPWV and Complior-cfPWV in Identifying Arteriosclerosis

Using cfPWV = 10 m/s as a CUT-OFF value, the consistency of HW-cfPWV and Complior-cfPWV in detecting arteriosclerosis (cfPWV > 10 m/s) is examined. The KAPPA value is 0.792, indicating that the two methods have good consistency in judging arteriosclerosis (cfPWV > 10 m/s), as shown in Table 3.

**TABLE 3:** Consistency of smartwatch and Complior arteriosclerosis analyzer in diagnosing arterial stiffness (HW cfPWV > 10 m/s vs. CopmilorcfPWV > 10 m/s).

			cfPWV measured with Complior arteriosclerosis analyzer		Total
			≤10 m/s	> 10 m/s	
cfPWV measured with smart watch	≤10 m/s	n	230	12	242
		Line percentage (%)	95.0	5.0	100.0
		Column percentage (%)	92.7	12.1	69.7
	> 10 m/s	n	18	87	105
		Line percentage (%)	17.1	82.9	100.0
		Column percentage (%)	7.3	87.9	30.3
Total		n	248	99	347
		Line percentage (%)	71.5	28.5	100.0
		Column percentage (%)	100.0	100.0	100.0

*Kappa = 0.792 and p < 0.001.*

### Error Analysis of HW-cfPWV and Complior-cfPWV

The MAE detected by HW-cfPWV was 0.657 (95% CI 0.587–0.726), RMSE was 0.927 (95% CI 0.806–1.035), and MAPE was 7.385% (95% CI 6.615% –8.154%). Although the error between the HW-cfPWV and the Complior-cfPWV measurement value is small, the error distribution is not normal, indicating that the error is affected by some non-random factors. From an individual perspective, 95% of the individual measurement error B-A is between −1.78 m/s to 1.87 m/s (Figure 3). The layered Bland-Altman analysis results show that as the age increases and the increase in shrinkage, the overall distribution range of the cfPWV is widened and dispersed, and the LOA of the ages of 60 years old is also wider, with a maximum can be ± 2 m/s, shown in Supplementary Figures 2, 3. According to the generalized linear model results, absolute errors have statistically significant associations with SBP and age, implying that age and blood pressure are independent factors influencing absolute error (Table 4).

**TABLE 4:** Multiple linear regression for influencing factors of absolute value error.

Variables	Unstandardized Coefficients		Standardized Coefficients	T value	*P* value	95% confidence interval	
	B	Standard error	Beta			lower	upper
Constants	0.536	0.305		1.759	0.080	−0.063	1.136
Age	0.008	0.002	0.229	3.945	0.000	0.004	0.012
DBP	−0.018	0.005	−0.254	−3.847	0.000	−0.027	−0.009
SBP	0.009	0.003	0.195	2.694	0.007	0.002	0.015

## Discussion

Arteriosclerosis is a vascular disease that worsens with age and increased risk factors (blood pressure and lipid levels) (11). Nowadays, young people have unhealthy lifestyles like high work pressure, high tension, less activity, smoking, and high salt intake (12, 13), resulting in increased arteriosclerosis and hypertension among them (14–16). Younger people are more likely to purchase smartwatches to better understand their physiological parameters. Active lifestyle intervention can help delay the progression of the vascular disease if signs of arteriosclerosis are detected early (17, 18).

The elasticity and stiffness of the elastic aorta, which are less affected by vasoactive substances in the blood, can be measured using cfPWV, and the results are repeatable. cfPWV is an important index for evaluating arterial stiffness that is currently accepted and recommended by domestic and international hypertension guidelines and early assessment content for arteriosclerosis caused by hypertension (8, 9). Increased arterial stiffness as a subclinical vascular disease is closely related to the occurrence and advancement of atherosclerotic cardiovascular disease (ASCVD) (1, 19) whereas cfPWV is a gold indicator for assessing arteriosclerosis (20, 21). However, at present, arteriosclerosis measurement devices are only used in hospitals. But it is not suitable for daily testing of families or individuals due to the large size of the equipment, the high cost of charges, and the relatively complicated operation.

Using mobile wearable devices has begun to develop active health models in the digital age. Mobile smartwatches can detect many physiological parameters, including heart rate, respiration, sleep, blood oxygen, and other parameters. These features are crucial in guiding active health for watch wearers. Many smartwatches can now measure ECG function and detect arrhythmia in real-time, allowing for atrial fibrillation screening (17, 22–25). Huawei designed the ECG + PPG of the watch to record the cardiac cycle and pulse wave *via* the smart terminal and developed the parameter of arterial elasticity estimation, namely cfPWV, using the actuarial calculation of machine learning. In this study, the Complior arteriosclerosis analyzer was used as the “gold standard” for cfPWV measurement, and the cfPWV estimated by smartwatches was compared with that of arteriosclerosis measurement instruments. The results of HW-cfPWV and Complior-cfPWV have high consistency (Kappa = 0.79) and diagnostic performance in judging arteriosclerosis (cfPWV > 10 m/s) area under the ROC curve 0.968, with sensitivity 0.899, specificity 0.927, positive predictive value 0.83, negative predictive value 0.96, macroscopic F1 value 86%. The results of multiple measurements and different body positions show that the smartwatch has good accuracy in measuring cfPWV, with a consistency correlation coefficient (CCC) of 0.9 and a mean absolute error of 0.66 m/s. The evaluation standard for the accuracy of non-invasive equipment for pulse wave velocity measurement is given based on the mean error (0.046 m/s) of two Bland-Altman analyses, according to the guidelines of the International Arterial Association (10) where cfPWV determination by HW smartwatch is ACCEPTABLE. This smart wearable device may aid in the early detection or prediction of arteriosclerosis.

However, a generalized linear model also showed a statistically significant association between age and systolic blood pressure. Bland-Altman stratification showed that the LoA was wider in those aged > 60 years and SBP ≥ 140 mmHg, indicating that age and blood pressure were the factors affecting the absolute value error. It is speculated that the reason may be due to age-related changes in arterial blood vessels. Peripheral arteriosclerosis is more common in the elderly and people with high blood pressure (26, 27). Radial arteriosclerosis can cause poor signal acquisition and changes in the pulse wave signal during the measurement of the watch, resulting in errors between the cfPWV value estimated by the smartwatch and the data measured by the Complior arteriosclerosis analyzer. Furthermore, the influencing factors of watching pulse waves are also related to measurement posture and dry skin. Once similar problems occur, the smartwatch system will remind the tester to repeat the measurement through the mobile APP and remind them to keep the arm flat and still during measurement or wipe the dry skin with alcohol to improve the signal quality. In this study, there were 130 elderly people, 38 of whom were older than 70 years, and 28 had systolic blood pressure greater than 140 mmHg. Statistical power may be underpowered due to the limited number of older adults and hypertensive patients with poorly controlled blood pressure. The results suggest that current smartwatch estimates of PWV, which can be applied to people aged < 60 and a systolic blood pressure < 140 mmHg. Further research on elderly people of different ages and people with various blood pressure levels should be conducted to better train the mathematical model into the watch to the corresponding population.

Smartwatches have a specific value in assessing vascular stiffness in young and middle-aged people. Our previous study (28) found that cfPWV had an upward trend in young and middle-aged people with high-normal blood pressure and no cardiovascular disease. Therefore, middle-aged people estimate arterial stiffness using smartwatches, which will help in the early detection of arteriosclerosis. Furthermore, smart terminal watches are more convenient and practical than large-scale professional equipment because they do not require professionals or complicated operations and provide a new and effective way for low-cost, large-scale arterial stiffness detection. The calculation function in the smartwatch and the corresponding APP on the mobile phone can help to promote early detection of arterial stiffness. When individuals wearing smartwatches have a trend of increasing cfPWV data at the same age, active lifestyle interventions (such as weight control, tobacco control, salt control, and so on) can be used to improve and delay the progression of arteriosclerosis through vibrant health. Notably, COVID-19 is still popular all over the world, which limits people’s outdoor activities to a certain extent. The application of intelligent wearable devices has become an assistant for monitoring health status. Smartwatches can monitor people’s health physiological parameters indoors or outdoors, obtaining basic health information for patients who cannot go out for activities or need hospital treatment due to the epidemic. This study shows that the Huawei smartwatch can accurately estimate cfPWV to evaluate arterial stiffness and help wearers assess their health status through health parameters. Therefore, it has a broader application prospect in today’s environment.

Limitations: The study found that the cfPWV obtained by current smartwatches has certain errors in older patients with elevated blood pressure. The current HW smartwatch for cfPWV determination is more suitable for self-monitoring of people aged < 60 years and SBP < 140 mmHg due to the limited population, particularly the number of patients older than 70 years old with hypertension and diabetes. However, it has some limitations as a medical diagnostic tool. Thus, it is recommended to go to the hospital for corresponding examinations if the smartwatch has repeatedly measured cfPWV for the same age or people with comorbidities. The study of using smartwatches to assess the accuracy of cfPWV in elderly and hypertensive patients is presently underway.

## Data Availability Statement

The raw data supporting the conclusions of this article will be made available by the authors, without undue reservation.

## Author Contributions

NS participated in the research design, guided the experiment process, and wrote the full text. LW participated in the research design, research test, and revision of the article. YX, HW, YYC, and JL participated in the revision of the article. FY and YXC participated in the study’s testing. ZZ was responsible for the statistical analysis of the data and the revision of the article. All authors contributed to the article and approved the submitted version.

## Conflict of Interest

The authors declare that the research was conducted in the absence of any commercial or financial relationships that could be construed as a potential conflict of interest.

## Publisher’s Note

All claims expressed in this article are solely those of the authors and do not necessarily represent those of their affiliated organizations, or those of the publisher, the editors and the reviewers. Any product that may be evaluated in this article, or claim that may be made by its manufacturer, is not guaranteed or endorsed by the publisher.

## References

[1] Yingchoncharoen T Limpijankit T Jongjirasiri S Laothamatas J Yamwong S Sritara P. Arterial stiffness contributes to coronary artery disease risk prediction beyond the traditional risk score (RAMA-EGAT score). *Heart Asia.* (2012) 4:77–82. 10.1136/heartasia-2011-010079 23585778 PMC3622433

[2] Otsuka K Fukuda S Shimada K Suzuki K Nakanishi K Yoshiyama M Serial assessment of arterial stiffness by cardio-ankle vascular index for prediction of future cardiovascular events in patients with coronary artery disease. *Hypertens Res.* (2014) 37:1014–20. 10.1038/hr.2014.116 25007768

[3] Yang XL Li JX Hu DS Chen J Li Y Huang J Predicting the 10-year risks of atherosclerotic cardiovascular disease in Chinese population: the China-PAR project (prediction for ASCVD risk in China). *Circulation.* (2016) 134:1430–40. 10.1161/CIRCULATIONAHA.116.022367 27682885

[4] Vasan RS Pan S Larson MG Mitchell GF Xanthakis V. Arteriosclerosis, atherosclerosis, and cardiovascular health: joint relations to the incidence of cardiovascular disease. *Hypertension.* (2021) 78:1232–40. 10.1161/HYPERTENSIONAHA.121.18075 34601961 PMC8516717

[5] Kim ED Ballew SH Tanaka H Heiss G Coresh J Matsushita K. Short-term prognostic impact of arterial stiffness in older adults without prevalent cardiovascular disease. *Hypertension.* (2019) 74:1373–82. 10.1161/HYPERTENSIONAHA.119.13496 31679417 PMC7110414

[6] Patel MS Asch DA Volpp KG. Wearable devices as facilitators, not drivers, of health behavior change. *JAMA.* (2015) 313:459–60. 10.1001/jama.2014.14781 25569175

[7] Wang L He SL Li B Guo JY Lei YH Liu Q Exploration of pulse wave transit time examination on the left wrist in hypertension patients and normal adults with smartwatch. *China Med Devicese.* (2017) 32:87–9.

[8] Williams B Mancia G Spiering W Agabiti Rosei E Azizi M Burnier M 2018 practice guidelines for the management of arterial hypertension of the European society of hypertension and the European society of cardiology: ESH/ESC task force for the management of arterial hypertension. *J Hypertens.* (2018) 36:2284–309. 10.1097/HJH.0000000000001961 30379783

[9] Chinese Hypertension League, Chinese Society of Cardiology, Hypertension Committee of the Chinese Medical Doctor Association, Hypertension Branch of the China Association for the Promotion of International Exchanges of Health Care, Hypertension Branch of the Chinese Geriatrics Society. 2018 Chinese guidelines for prevention and treatment of hypertension-a report of the revision committee of Chinese guidelines for prevention and treatment of hypertension. *J Geriatr Cardiol.* (2019) 16:182–241. 10.11909/j.issn.1671-5411.2019.03.014 31080465 PMC6500570

[10] Wilkinson IB McEniery CM Schillaci G Boutouyrie P Segers P Donald A ARTERY society guidelines for validation of non-invasive haemodynamic measurement devices: part 1, arterinal pulse wave velocity. *Artery Res.* (2010) 4:34–40. 10.1016/j.artres.2010.03.001

[11] Climie RE Bruno RM Hametner B Mayer CC Terentes-Printzios D. Vascular age is not only atherosclerosis, it is also arteriosclerosis. *J Am Coll Cardiol.* (2020) 76:229–30. 10.1016/j.jacc.2020.03.081 32646575

[12] Zhang J Guo X Lu Z Tang J Li Y Xu A Cardiovascular diseases deaths attributable to high sodium intake in Shandong province, China. *J Am Heart Assoc.* (2019) 8:e010737. 10.1161/JAHA.118.010737 30563415 PMC6405719

[13] The Writing Committee of the Report on Cardiovascular Health and Diseases in China. Key points of report on cardiovascular health and diseases in China 2020. *Chin J Cardiovasc Res.* (2021) 7:582–90.

[14] Zhang Y Lacolley P Protogerou AD Safar ME. Arterial stiffne in hypertension and function of large arteries. *Am J Hypertens.* (2020) 33:291–6. 10.1093/ajh/hpz193 32060496

[15] Ahammed B Maniruzzaman M Talukder A Ferdausi F. Prevalence and risk factors of hypertension among young adults in Albania. *High Blood Press Cardiovasc Prev.* (2021) 28:35–48. 10.1007/s40292-020-00419-5 33113094

[16] Liu J Lu XZ Chen LY Huo YI. Expert consensus on the management of hypertension in the young and middle-aged Chinese population. *Int J Clin Pract.* (2019) 73:e13426. 10.1111/ijcp.13426 31573725

[17] Guo Y Wang H Zhang H Liu T Liang Z Xia Y Mobile photoplethysmographic technology to detect atrial fibrillation. *J Am Coll Cardiol.* (2019) 74:2365–75. 10.1016/j.jacc.2019.08.019 31487545

[18] Doughty KN Del Pilar NX Audette A Katz DL. Lifestyle medicine and the management of cardiovascular disease. *Curr Cardiol Rep.* (2017) 19:116. 10.1007/s11886-017-0925-z 28980137

[19] Alonso-Domínguez R Sánchez-Aguadero N Patino-Alonso MC Agudo-Conde C de Cabo-Laso Á Gómez-Sánchez M Association between measurements of arterial stiffness and target organ damage in a general Spanish population. *Ann Med.* (2021) 53:345–56. 10.1080/07853890.2021.1881812 33533280 PMC7877984

[20] Van Bortel LM Laurent S Boutouyrie P Chowienczyk P Cruickshank JK De Backer T Expert consensus document on the measurement of aortic stifnesindaily practice using carotid-femoral pulse wave velocity. *J Hypertens.* (2012) 30:445–8. 10.1097/HJH.0b013e32834fa8b0 22278144

[21] Milan A Zocaro G Leone D Tosello F Buraioli I Schiavone D Current assessment of pulse wave velocity: comprehensive review of validation studies. *J Hypertens.* (2019) 37:1547–57. 10.1097/HJH.0000000000002081 30882597

[22] Guo YT Wang H Zhang H Chen Y Lip GYH. Population-based screening or targeted screening based on initial clinical risk assessment for atrial fibrillation: a report from the Huawei heart study. *J Clin Med.* (2020) 9:1493. 10.3390/jcm9051493 32429241 PMC7291296

[23] Chen ED Jiang J Su R Gao M Zhu SN Zhou J A new smart wristband equipped with an artificial intelligence algorithm to detect atrial fibrillation. *Heart Rhythm.* (2020) 17(5 Pt B):847–53. 10.1016/j.hrthm.2020.01.034 32354449

[24] Bumgarner JM Lambert CT Hussein AA Cantillon DJ Baranowski B Wolski K Smartwatch algorithm for automated detection of atrial fibrillation. *J Am Coll Cardiol.* (2018) 71:2381–8. 10.1016/j.jacc.2018.03.003 29535065

[25] Rajakariar K Koshy AN Sajeev JK Nair S Roberts L Teh AW. Accuracy of a smartwatch based single-lead electrocardiogram device in detection of atrial fibrillation. *Heart.* (2020) 106:665–70. 10.1136/heartjnl-2019-316004 31911507

[26] Isobe T Saitoh S Takagi S Ohnishi H Ohhata J Takeuti H Relation of hypertension and glucose tolerance impairment in elderly people to the development of arteriosclerosis–investigation using pulse wave velocity. *Nihon Ronen Igakkai Zasshi.* (2003) 40:610–4. 10.3143/geriatrics.40.610 14689853

[27] Sun Z. Aging, arterial stiffness, and hypertension. *Hypertension.* (2015) 65:252–6. 10.1161/HYPERTENSIONAHA.114.03617 25368028 PMC4288978

[28] Wang LY Sun NL Wu YT Wu SL Liu XY. The changes of arterial stiffness and its influencing factors in overweight population with abdominal obesity and high-normal blood pressure. *Chin J Hypertens.* (2020) 28:65–9. 10.16439/j.cnki.1673-7245.2020.01.020

