# Supplementary material for: Accuracy Evaluation of Carotid-Femoral Pulse Wave Velocity Estimated by Smart Terminal Watch
Source: Front Cardiovasc Med. 2022 Jul 22;9:893557. doi: 10.3389/fcvm.2022.893557 (PMC9353553; doi:10.3389/fcvm.2022.893557)
Supplement: Supplementary file 1 [file Data_Sheet_1.docx]

**Supplementary materials**

Suppl Table 1. Stratified analysis for correlation coefficient between HW-cfPWV and Complior-cfPWV

|  |  | correlation coefficient * | P value |
| --- | --- | --- | --- |
| Total (n=347) |  | 0.9182 | <0.0001 |
| age | <30 (n=103) | 0.8629 | <0.0001 |
|  | 30-60 (n=108) | 0.8757 | <0.0001 |
|  | >60 (n=136) | 0.8541 | <0.0001 |
| risk factors | No (n=243) | 0.9223 | <0.0001 |
|  | With (n=104) | 0.8472 | <0.0001 |
| medication | No (n=247) | 0.9273 | <0.0001 |
|  | With (n=100) | 0.8374 | <0.0001 |
| Sysdolic blood pressure | <140mmHg (n=319) | 0.9076 | <0.0001 |
|  | ≥140mmHg (n=28) | 0.9295 | <0.0001 |
| Heart rate | <80bpm (n=300) | 0.9169 | <0.0001 |
|  | ≥80bpm (n=47) | 0.9309 | <0.0001 |

* between HW-cfPWV and Complior-cfPWV

## Suppl Table 2. Paired T-Test for equivalence using TOST (Two one-sided tests)

| Equivalence hypothesis: -1 m/s < (mean of difference between Complior cfPWV and HW-cfPWV)< 1 m/s | | | | | | | |
| --- | --- | --- | --- | --- | --- | --- | --- |
|  | Alternative | Mean | Standard |  |  | Prob | Reject H0 |
| Test | Hypothesis† | Difference | Error | T-Statistic | DF | Level | at α = 0.050? |
| Lower Boundary | Diff > -1 | -0.04607758 | 0.04979398 | 19.1574 | 346 | 0.00001 | Yes |
| Upper Boundary | Diff < 1 | -0.04607758 | 0.04979398 | -21.0081 | 346 | 0.00001 | Yes |
| Equivalence | -1 < Diff < 1 | -0.04607758 |  |  |  | 0.00001 | Yes |

† "Diff" refers to the Mean of the Paired Differences. Power = 0.99

## Suppl Table 3. Wilcoxon rank sum test based on TOST (Two one-sided tests)

| Test | Sum of Ranks (W) | Mean of W | Std Dev of W | Number of Zeros | Number of Sets Ties | Multiplicity Factor |
| --- | --- | --- | --- | --- | --- | --- |
| Lower Boundary | 35151 | 30189 | 1869.998 | 0 | 0 | 0 |
| Upper Boundary | 21869 | 30189 | 1869.998 | 0 | 0 | 0 |

Equivalence hypothesis: -0.15 < (Median of difference between Complior cfPWV and HW-cfPWV)< 0.15


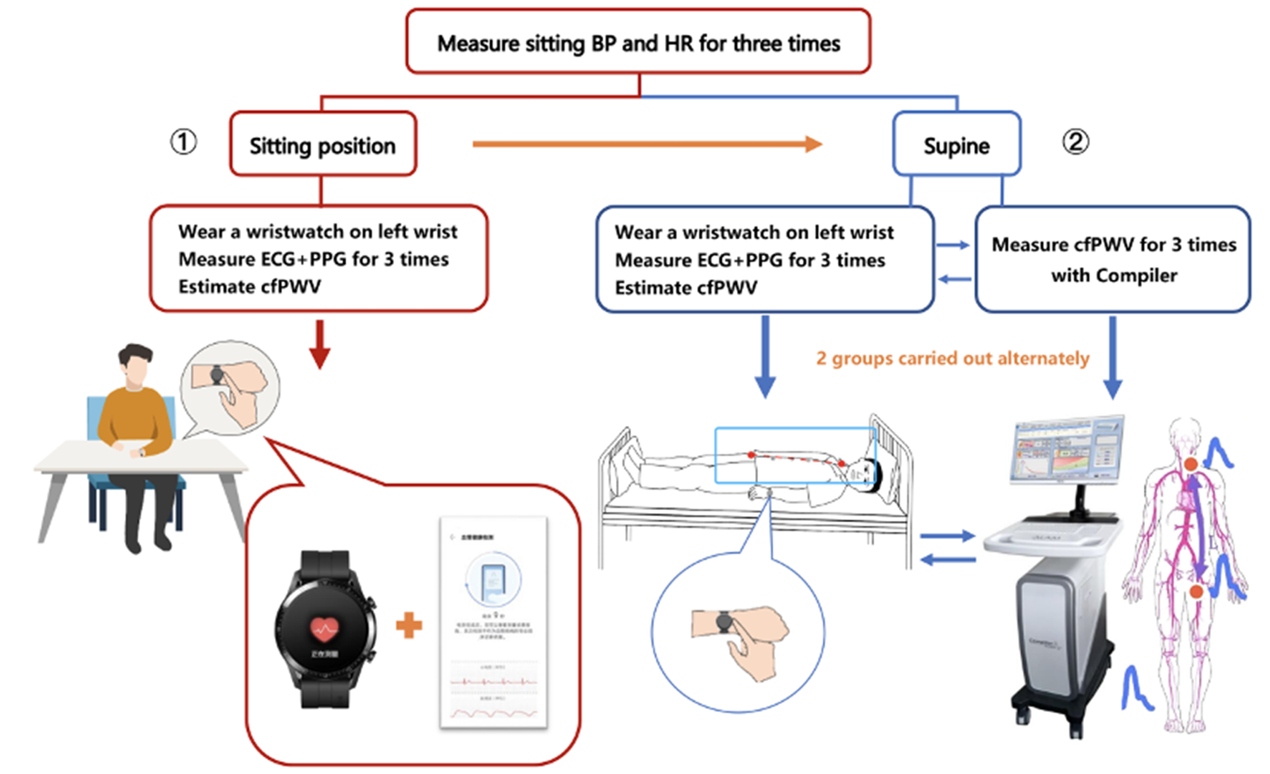


Suppl Figure 1. Flowcharts of two methods to measure cfPWV. Note: ① First step: measure cfPWV with an intelligent terminal watch for three times in sitting position; ② Second step：measure cfPWV with smart watch and Complier equipment three times alternately in the supine position.


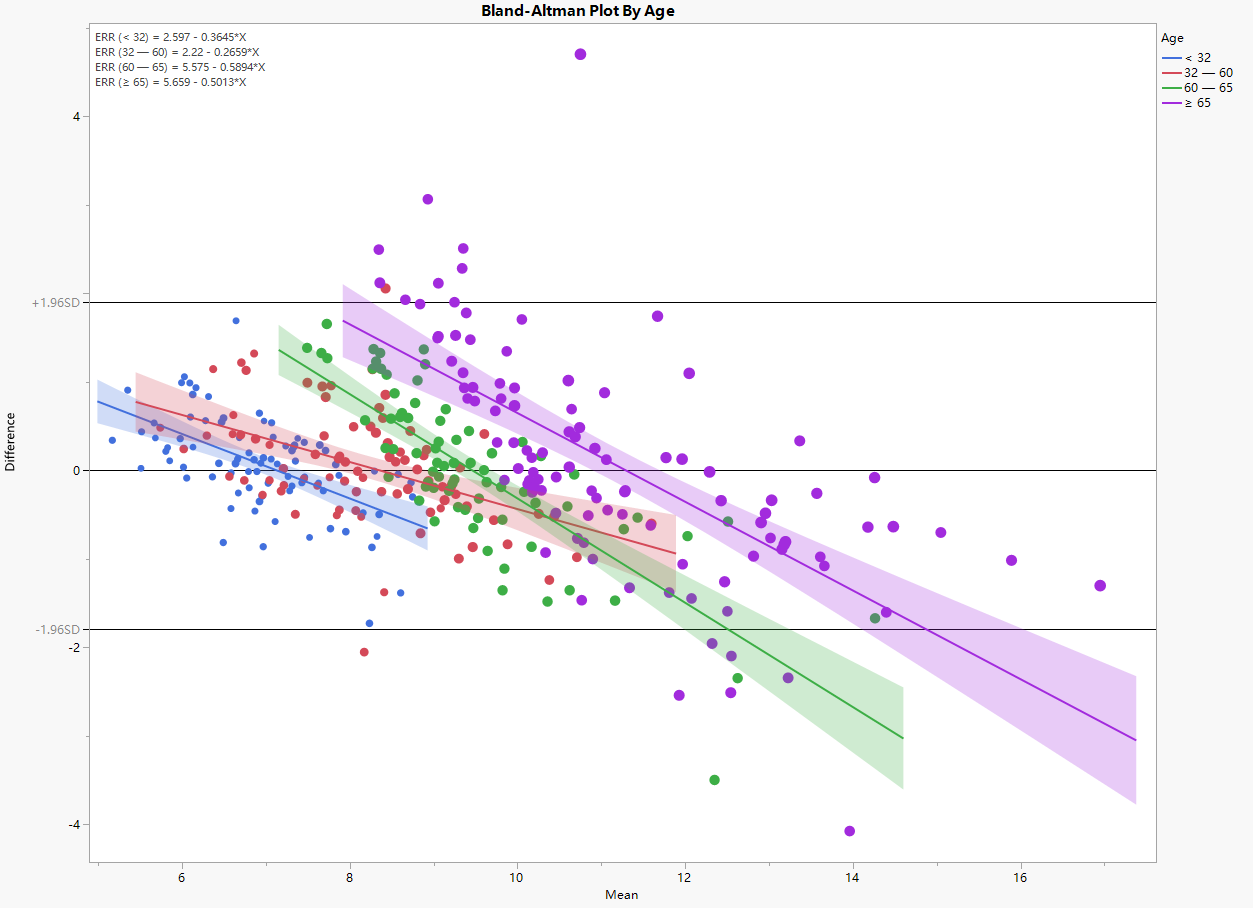


Suppl Figure 2. Bland Altman analysis of errors at different ages.


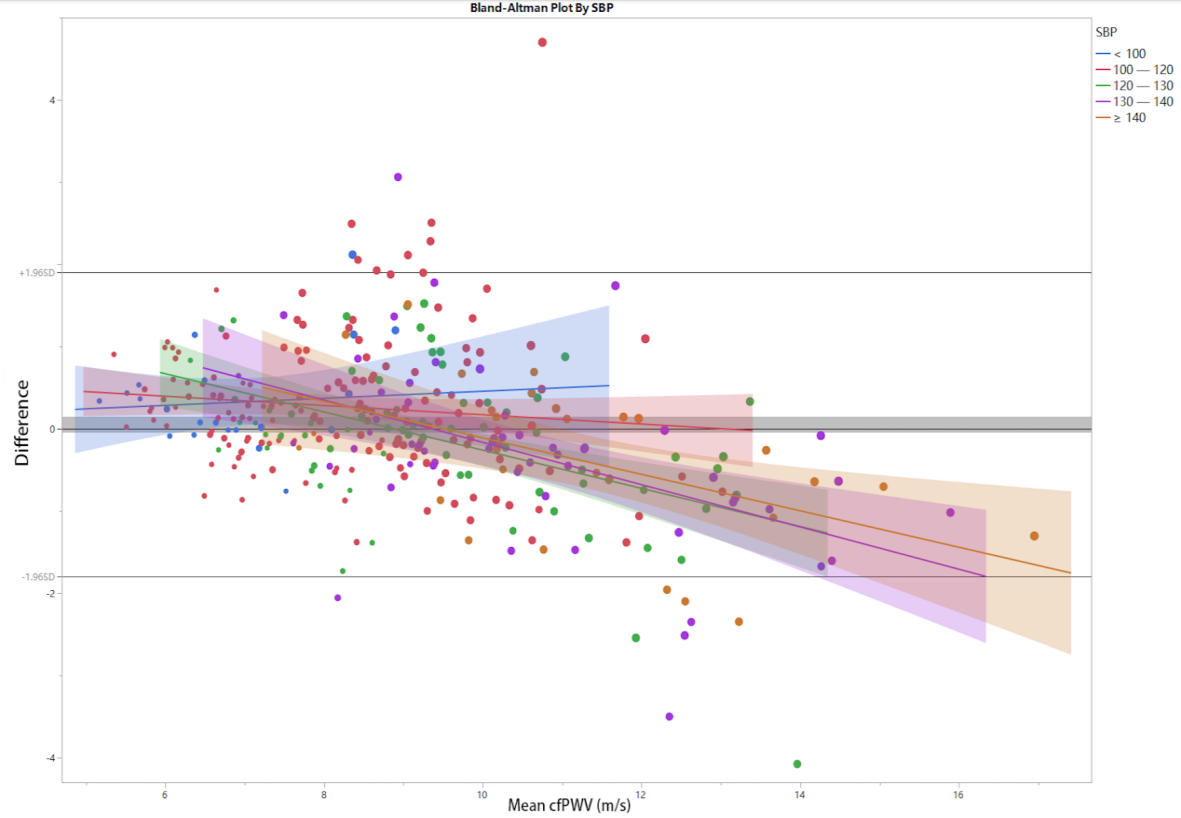


Suppl Figure 3. Bland Altman analysis of errors at different blood pressures.
